# Effect of Ionic Group on the Complex Coacervate Core Micelle Structure

**DOI:** 10.3390/polym11030455

**Published:** 2019-03-10

**Authors:** Tae-Young Heo, Inhye Kim, Liwen Chen, Eunji Lee, Sangwoo Lee, Soo-Hyung Choi

**Affiliations:** 1 Department of Chemical Engineering, Hongik University, Seoul 04066, Korea; tyheo@mail.hongik.ac.kr; 2 Graduate School of Analytical Science and Technology, Chungnam National University, Daejeon 34134, Korea; inhyekim@cnu.ac.kr; 3 Department of Chemical & Biological Engineering, Rensselaer Polytechnic Institute, Troy, NY 12180, USA; chenl15@rpi.edu (L.C.); lees27@rpi.edu (S.L.); 4 School of Materials Science and Engineering, Gwangju Institute of Science and Technology, Gwangju 61005, Korea; eunjilee@gist.ac.kr

**Keywords:** complex coacervate core micelle, ionic group, small-angle neutron scattering (SANS), contrast matching technique

## Abstract

Pairs of ionic group dependence of the structure of a complex coacervate core micelle (C3M) in an aqueous solution was investigated using DLS, cryo-TEM, and SANS with a contrast matching technique and a detailed model analysis. Block copolyelectrolytes were prepared by introducing an ionic group (i.e., ammonium, guanidinium, carboxylate, and sulfonate) to poly(ethylene oxide-*b*-allyl glycidyl ether) (*N*_PEO_ = 227 and *N*_PAGE_ = 52), and C3Ms were formed by simple mixing of two oppositely-charged block copolyelectrolyte solutions with the exactly same degree of polymerization. All four C3Ms are spherical with narrow distribution of micelle dimension, and the cores are significantly swollen by water, resulting in relatively low brush density of PEO chains on the core surface. With the pair of strong polyelectrolytes, core radius and aggregation number increases, which reflects that the formation of complex coacervates are significantly sensitive to the pairs of ionic groups rather than simple charge pairing.

## 1. Introduction

When oppositely-charged polyelectrolyte solutions are mixed, a liquid-liquid phase separation occurs that is called “complex coacervation” [1,2,3]. Complex coacervates, polymer-rich domains, show good hydrophilicity due to significantly large amount of water inside the domain, and low interfacial tension [4,5]. In addition, complex coacervates are highly responsive to internal and external stimuli such as pH, salt concentration, molecular weight, and charge density, because the phase separation is driven by electrostatic interaction between oppositely-charged polyelectrolytes, and entropic gain of counter ion release. Therefore, the complex coacervates have been widely applied in the field of cosmetics, biomedical engineering, and the food industry [6,7,8,9,10,11,12,13,14,15].

Analogous to traditional block copolymer micelles, double hydrophilic block copolyelectrolytes can self-assemble into micelles in an aqueous solution by mixing with oppositely-charged polyelectrolytes, which are called “complex coacervate core micelles” (C3Ms). Typically, C3Ms are composed of a complex coacervate core of polyelectrolyte block and a swollen corona of soluble blocks (e.g., PEO) [11,16,17,18,19]. Parallel to the bulk complex coacervates, C3Ms show relatively hydrophilic core and great stimuli-responsiveness to pH and salt concentration [20,21,22,23,24,25,26]. In addition, C3Ms form nanometer-sized structure in a controlled fashion, and coacervate cores are shielded by corona blocks. With these advantages, C3Ms have been widely employed as delivery vehicles for DNA, RNA, and protein, or contrast agents [20,21,22,24,25,26,27,28,29,30].

Most investigations dealing with C3Ms have attempted to employ the combination of block copolyelectrolytes (BCPs) and homopolyelectrolytes (HPs) due to large applicability by mimicking bio-inspired nature and limited synthetic techniques [16,17,21,22,28,31,32,33,34,35,36,37,38]. However, investigation on C3Ms formed by two oppositely-charged BCPs is necessary to understand and control thermodynamics and dynamics underlying C3Ms, because HPs usually have large degree of freedom, and are difficult to manage. In contrast to C3Ms of BCP + HP, Harada and Kataoka reported the chain length recognition where C3Ms are formed only when the length of charged block is matched for two oppositely-charged BCPs [39]. In addition, Koide et al. reported that the C3Ms of two oppositely-charged BCPs form vesicles, which follows the strategy of the traditional block copolymer micelles [27]. With the C3Ms of two BCPs, highly-ordered and better controlled structures, therefore, can be achieved. However, much less is known about the detailed characteristics and morphology of C3Ms as a function of charged functional group, salt concentration, pH and block ratio yet.

In this study, the effect of the pair of ionic groups on the structure of C3Ms is investigated using DLS, cryo-TEM, and SANS. We synthesized well-defined block copolyelectrolytes by functionalizing a parent block copolymer using thiol-ene click chemistry to eliminate side effects including the charge imbalance and backbone length mismatch between two BCPs. Four ionic groups were employed which are categorized as a weak ionic group (e.g., ammonium (A) and carboxylate (C)) and a strong ionic group (e.g., guanidinium (G) and sulfonate (S)) depending on the pKa values. The mixing of two oppositely-charged BCPs with ionic groups including A + S, A + C, G + S, and G + C provides C3Ms with different nature of the coacervate cores. Our finding shed a new insight on the emerging and technologically important topic of complex coacervates-driven structures in an aqueous solution.

## 2. Materials and Methods

### 2.1. Materials

**Figure 1:**
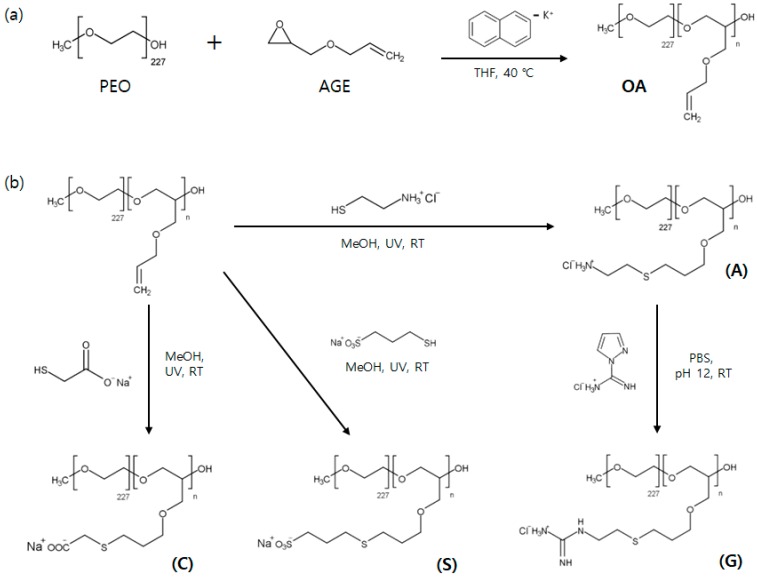
Synthetic scheme of (**a**) OA diblock copolymers using anionic ring-opening polymerization, and (**b**) post-modification of allyl group by thiol-ene “click” chemistry to introduce functional group of ammonium (A), guanidinium (G), carboxylate (C), and sulfonate (S).

Poly(ethylene oxide-*b*-allyl glycidyl ether) (OA) diblock copolymers were synthesized by anionic ring-opening polymerization, and followed by post-modification to introduce a charged group to AGE block [40,41]. (See Figure 1) Allyl glycidyl ether (AGE, TCI, Tokyo, Japan) was degassed via several freeze-pump-thaw cycles, and purified with butyl magnesium chloride for 2 h. Tetrahydrofuran (THF) was used as a solvent and collected from a dry solvent system. Potassium naphthalenide was prepared by mixing potassium metal and naphthalene in dry THF for at least 24 h. Poly(ethylene oxide) (PEO-CH_3_, *M*_n_ = 10 kg/mol, Sigma-Aldrich, St. Louis, MO, USA) was purchased and used as received. Deuterated poly(ethylene oxide) (*M*_n_ = 10 kg/mol) was synthesized using deuterated ethylene oxide (Polymer Source) initiated by *sec*-butyl lithium.

Normal and deuterated poly(ethylene oxide) was dried for at least 24 h under vacuum to remove residual water, and used as a macroinitiator. Under an argon atmosphere, PEO in dry THF was initiated by addition of potassium naphthalenide solution until slight green color appears. Then, AGE monomers were added and polymerized for at least 24 h at 40 °C [42]. Degassed methanol was injected into the reactor to terminate the polymerization. OA and dOA diblock copolymers were then recovered by precipitation in *n*-hexane.

Either cationic and anionic groups were introduced to allyl group of AGE block by thiol-ene click chemistry [40]. OA (or dOA) diblock copolymers and 2,2-dimethyoxy-2-phenylacetophenone (DMPA, 0.05 equiv. per alkene group), a photoinitiator, were dissolved in methanol, and a functional thiol reagent such as cysteamine hydrochloride (Sigma Aldrich, St. Louis, MO, USA), sodium 3-mercapto-1- propanesulfonate (Sigma Aldrich), and thioglycolic acid (Sigma Aldrich)) was dissolved in distilled water separately (3 equiv. per alkene group). Mixture of the two solutions were purged by argon for 10 min, and followed by exposure to UV light with stirring for at least 3 h. Residuals were removed by dialysis with regenerated cellulose dialysis tubing (Fisher Scientific, MWCO 3500, Waltham, MA, USA) in ultra-high-purity water. Then, the lyophilization was carried out for 3 days. Guanidinium functional group is introduced by mixing ammonium functionalized block copolymer with 1H-Pyrazole-1-carboxamidine hydrochloride (Sigma Aldrich) in PBS buffer solution (pH ~ 12) for at least 4 days, and followed by dialysis and lyophilization. Finally, ammonium(A)-, guanidinium(G)-, carboxylate(C)-, and sulfonate(S)-functionalized OA and dOA block copolyelectrolytes were obtained.

Polymers were characterized by size exclusion chromatography (SEC, JASCO, Tokyo, Japan) and ^1^H nuclear magnetic resonance spectroscopy (^1^H NMR, Unity-Inova 500, Palo Alto, CA, USA). SEC was used to measure the molecular weight of dPEO homopolymer and the overall polydispersity index (*M*_w_/*M*_n_) of OA and dOA diblock copolymers. Molecular weight of AGE block was determined by the combination of ^1^H NMR spectroscopy and the molecular weight of the initiated PEO block. Molecular weight of AGE block in dOA polymer is estimated by the SEC traces. (See Figure S1) In addition, the extent of ionic functionalization for AGE block was measured by ^1^H NMR in D_2_O (~100%). (See Figures S2 and S3) Molecular characteristics of OA and dOA diblock copolyelectrolytes are provided in Table 1.

Polymer solutions were prepared by simple mixing of two oppositely-charged block copolymers in an aqueous solution. Positively- and negatively-functionalized OA diblock copolyelectrolytes were dispersed in an aqueous solution separately, and followed by mixing two solutions under stirring for at least 10 min.

### 2.2. Dynamic Light Scattering (DLS)

DLS measurements were performed using a Brookhaven BI-200SM goniometer (Long Island, NY, USA) with λ = 639 nm. The intensity autocorrelation function was measured at an angle of 90°, and temperature was maintained at 25 °C. Polymer solutions were loaded in a disposable borosilicate glass cell after passing through a syringe filter (0.45 μm pore size) to remove unexpected large particles including dust.

The intensity autocorrelation function, *g*^(2)^(*q*,*t*), was reproduced by the cumulant method to obtain an average decay rate (*Γ*) and the second cumulant (*μ*_2_) [43]. Corresponding mean hydrodynamic radius (*R*_h_) was then calculated using the Stokes-Einstein equation, *R*_h_ = *kT* / 6π*η*_s_*D*_0_, where *k*, *T*, *η*_s_, and *D*_0_ (=*Γ*/*q*^2^, where *q* = 4*πnλ*^−1^sin(θ/2) is the scattering wave vector and *n* is the refractive index of the solvent) are the Boltzmann constant, absolute temperature, solvent viscosity, and diffusion coefficient, respectively. The relative width of the distribution in the decay rate is estimated as *μ*_2_/*Γ*^2^. In addition, the distribution of *R*_h_ were extracted from the decay rate distributions obtained through the inverse Laplace transform program REPES [44].

### 2.3. Cryogenic Transmission Electron Microscopy (Cryo-TEM)

Cryo-TEM experiments were performed using JEM-1400 TEM (Peabody, MA, USA) operating at 120 kV and the electron micrographs were recorded on a side-mounted 2k × 2k Veleta CCD camera. Cryo-TEM samples were prepared in a home-built environmental chamber with 97–99% humidity at room temperature to prevent water evaporation of samples [45]. Micelles solutions (~3 μL) were transferred onto a lacey carbon-supported TEM grid, and the excess solution was blotted with a filter paper for 2–3 sec to form thin films of 100–300 nm thickness. Then, the samples were vitrified by plunging into liquid ethane at its melting temperature (~–183 °C) and the vitrified samples were mounted on a cryogenic sample holder. The data were analyzed with an image processing software RADIUS (Olympus Soft Imaging Solutions GmbH, NRW, Germany).

### 2.4. Small Angle Neutron Scattering (SANS)

SANS measurement was performed at 40 m-SANS (QUOKKA) at Australian Nuclear Science and Technology Organization (ANSTO) using a neutron wavelength of 6 Å [46]. The sample-to-detector distance was chosen as 1.35 and 10.04 m to provide *q* range of 0.005 Å^−1^ < *q* < 0.48 Å^−1^. Two-dimensional isotropic data were corrected for detector sensitivity, sample transmission, empty cell scattering, and sample thickness and then azimuthally averaged. The scattering intensity was reduced to absolute scale by the direct beam flux method. The coherent scattering intensity was obtained by subtraction of the solvent scattering.

Sample specimens of 0.5 wt % deuterated micelle solution in an isotopic mixture of D_2_O and H_2_O were loaded in quartz cells with ca. 1 mm or 2 mm thickness. Scattering data were collected for at least 20 min at 25 °C. Based on the densities of solvents and polymers, the scattering length density (SLD) were calculated as 6.43 × 10^−6^ Å^−2^, –0.56 × 10^−6^ Å^−2^, and 6.39 × 10^−6^ Å^−2^ for dPEO, H_2_O, and D_2_O, respectively. SLD of the coacervate core was experimentally determined as 1.31 × 10^−6^ Å^−2^, 1.34 × 10^−6^ Å^−2^, 1.55 × 10^−6^ Å^−2^ and 1.75 × 10^−6^ Å^−2^ for A + S, A + C, G + S, and G + C cores, respectively, which shows good agreement with the previous reports [41,47]. (See Figure S4 and S5, and Table S1) For the contrast matching experiments on these functionalized OA micelles, SLD of the isotopic solvent mixture was exactly matched to either SLD of corona (i.e., core contrast system) or SLD of core (i.e., corona contrast system). SLD of the isotopic solvent mixture was modulated by SLD_solvent_ = *ϕ*_H2O_ SLD_H2O_ + (1 − *ϕ*_H2O_) SLD_D2O_, where *ϕ*_H2O_ is the volume fraction of H_2_O in the solvent mixture.

The reduced SANS data were adjusted with a detailed fitting model where micelles are modeled as spherical cores with polydisperse core radii and polymer brushes attached to the core surface following spline *b* function. The mathematical form is described in the Appendix A. In all, nine parameters could be adjusted in the fits: the core radius (*R*_core_), the width of the distribution for the core radius (*σ*_core_), the radius of gyration of the corona chains (*R*_g_), the width of the core-corona interface (*σ*_int_), two terms (*a*_1_, *s*) in the cubic *b* spline function for the corona, the effective hard sphere radius (*R*_hs_), the hard sphere volume fraction (*ϕ*_hs_), and the aggregation number (*N*_agg_).

## 3. Results and Discussion

### 3.1. General Consideration

**Figure 2:**
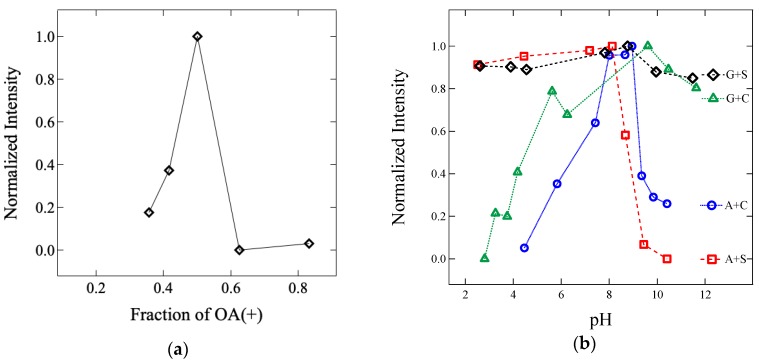
Normalized light scattering intensity with an angle of 90° as a function of (**a**) the fraction of cationic block copolyelectrolyte of G + S at pH of 7 and (**b**) pH of the solution at 1:1 mole ratio of A + S (square, red), A + C (circle, blue), G + S (diamond, black), and G + C (triangle, green) in an aqueous solution (color online).

Complex coacervate core micelles (C3M) are prepared by simple mixing of oppositely-charged block copolyelectrolyte solutions when the charge is stoichiometrically balanced at 1:1 mole ratio [16,23,36]. Figure 2a shows the normalized intensity of light scattering with the angle of 90° as a function of the fraction of positively charged block copolyelectrolyte in the G + S C3M solution. Since the light scattering intensity increases as the molecular weight of the nano-sized aggregates increases in a transparent solution, maximum C3Ms are formed upon charge matching at a 1:1 mole ratio of cationic and anionic block copolyelectrolytes.

In addition to the number of molecules, pH of the aqueous solution affects the degree of ionization for the charged block, and thus, the formation of C3M. Lower pH value than pKa of the negatively charged groups leads to form a neutral block of S and C, where a hydrogen atom is bonded to the moiety. Similarly, higher pH values than pKa of the positively-charged moiety results in a neutral block of G and A. Therefore adjusted solution pH between two limits is required to achieve nearly full ionization of the charged group, which results in charge stoichiometric balance, and thus the formation of C3M. Figure 2b displays the normalized intensity of light scattering at 90° as a function of solution pH for A + S, A + C, G + S, and G + C C3Ms at 1:1: mole ratio. Since pKa value is known as nearly 1, 4, 11, and 14 for S, C, A, and G, respectively [48,49,50], stable C3Ms are formed between two pKa of the pairs of charged groups, which shows good agreement with the previous reports [18,22]. For example, G + S C3Ms are stable between pH of 2 and 12, but A + S C3Ms become unstable above pH of 9 due to deprotonation of ammonium group [22,30]. Based on the condition for charge stoichiometric balance, the pH of each C3M solution was adjusted to 5, 8, 5, and 9 for A + S, A + C, G + S, and G + C, respectively, by addition of HCl or NaOH.

### 3.2. DLS & Cryo-TEM

**Figure 3:**
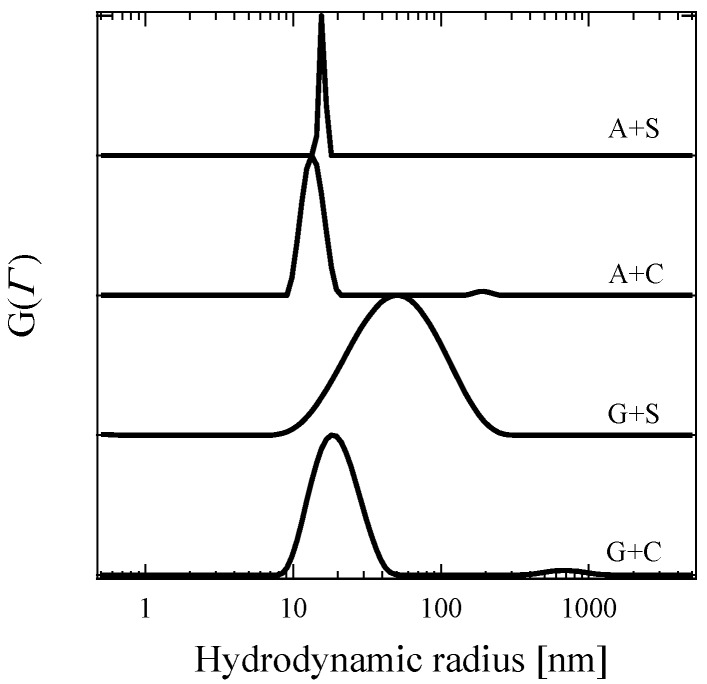
Distribution of hydrodynamic radius of C3Ms for A + S, A + C, G + S, and G + C at the scattering angle of 90°. Data are vertically shifted for convenience.

Figure 3 displays the distribution of hydrodynamic radii (*R*_h_) obtained from 0.5 wt % C3Ms for A + S, A + C, G + S, and G + C in an aqueous solution at the optimized pH using REPES method. In addition to the REPES method, the cumulant method provides averaged *R*_h_ (and the width of the distribution in *R*_h_) which is 16 (0.06), 14 (0.06), 40 (0.40), and 18 (0.14) nm for A + S, A + C, G + S, and G + C, respectively. Both methods provide narrow distribution in *R*_h_ for A + C and A + S, which reflects monodispersed spherical micelles. It is, however, suspected that broad distribution for G + C and G + S may result from micelle morphology other than spheres or micelle aggregates [27,32,51,52,53,54,55].

**Figure 4:**
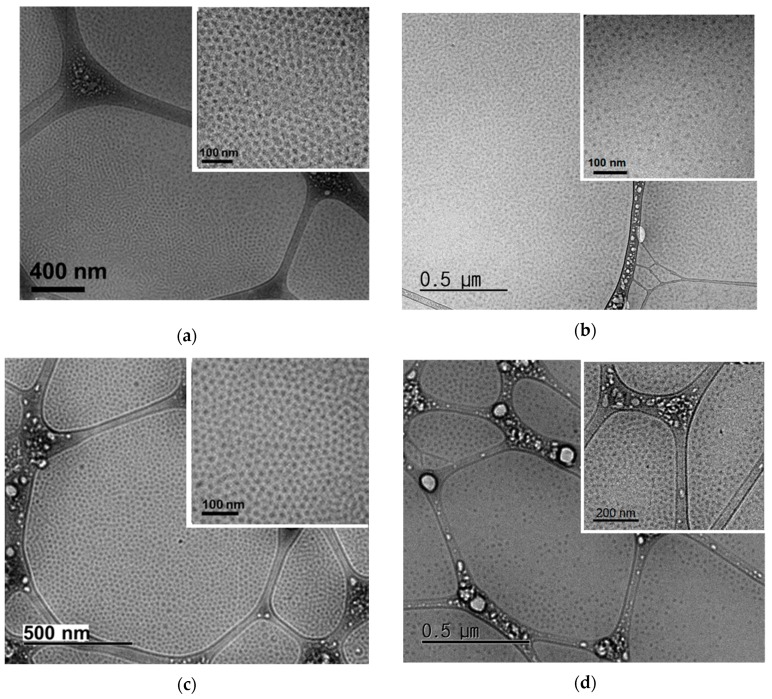
Cryo-TEM images obtained from 0.5 wt % C3Ms for (**a**) A + S; (**b**) A + C; (**c**) G + S; and (**d**) G + C in an aqueous solution. Insets are images of high resolution.

Cryo-TEM was used to image the morphology of the 0.5 wt % C3Ms in an aqueous solution with respect to the pair of ionic groups (i.e., A + S, A + C, G + S, and G + C) in Figure 4, which indicates that all four C3Ms are spherical (Note that cryo-TEM micrographs are obtained from 0.5 wt % block copolyelectrolytes micelles in aqueous solution that was based on OA with *N*_PEO_ = 227 and *N*_PAGE_ = 71. Since OA block copolymer (*N*_PEO_ = 227 and *N*_PAGE_ = 52) in this study shows smaller core block, spherical morphology should be retained [27,51]). It is interesting that 0.5 wt % C3M solutions form highly concentrated micelles within a thin layer on TEM grid. This is mainly due to the sample preparation process where a filter paper blotted water significantly more than C3Ms [47]. Therefore, all C3Ms in this study form spherical micelles, and thus, a non-spherical morphology for G + S and G + C C3Ms can be ruled out.

Previously, when C3Ms are formed, micellar aggregates have been proposed with open questions. Yusa et al. reported that oppositely-charged block copolyelectrolytes can form intermicellar aggregates in an aqueous solution. They employed the combination of dynamic/static light scattering (DLS/SLS) measurements and scanning electron microscopy (SEM) to investigate C3Ms formed by a mixture of oppositely-charged PEG-*b*-PMAPTAC (i.e., cationic block copolyelectrolyte) and PEG-*b*-PAMPS (i.e., anionic block copolyelectrolyte). Based on the significantly large *R*_h_ and spherical shape in the SEM, multicore intermicellar aggregates were speculated [54]. In addition, Voets et al. reported that the small fraction of larger aggregates such as cluster of micelles can exist when C3Ms are formed [36]. They investigated C3Ms formed by a mixture of PAA-*b*-PAAm (i.e., anionic block copolyelectrolyte) and P2MVP (i.e., cationic polyelectrolyte) in an aqueous solution, and observed upturns at low *q* in small-angle neutron scattering (SANS) profile, which may be due to micellar aggregates. Furthermore, van der Kooij et al. reported the possible presence of larger aggregates when C3Ms are formed by PEO-*b*-PM2VP (i.e., cationic block copolyelectrolyte) and PAA (i.e., anionic polyelectrolyte) [32]. They also observed slight upturn at low *q* in small-angle X-ray scattering (SAXS) profile for spherical C3Ms. Therefore, we speculated that broad distribution in *R*_h_ observed from 0.5 wt % C3Ms of G + S and G + C may be explained by aggregates of spherical micelles. For more detailed structure of the spherical micelles, small-angle X-ray/neutron scattering (SAX/NS) was performed [41,56,57,58]. (Note that SAXS analysis is described in Supporting Information, where the SAXS profiles are reproduced by the core-shell sphere model in Figure S6 and Table S2)

### 3.3. Small-Angle Neutron Scattering (SANS)

**Figure 5:**
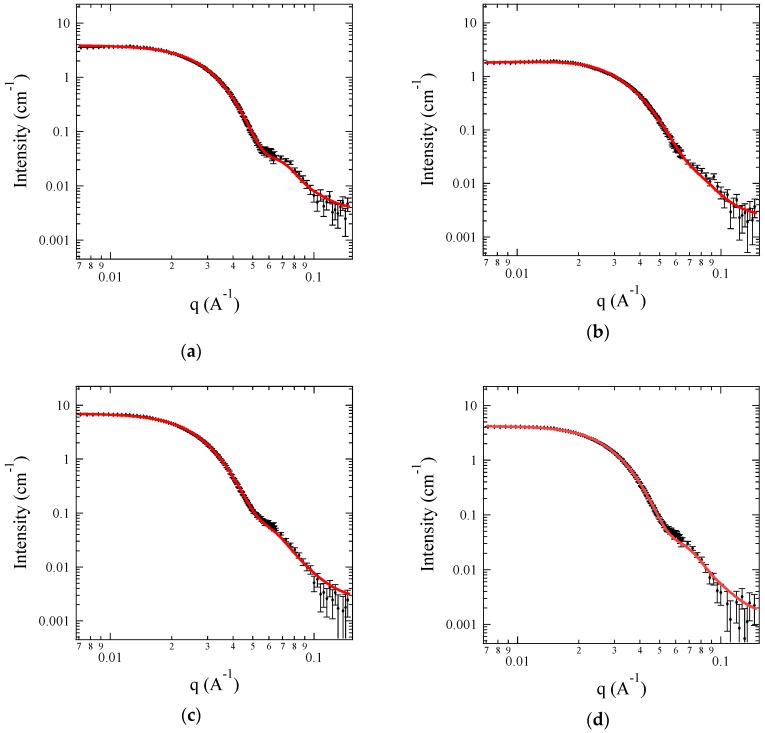
SANS profiles of 0.5 wt % C3Ms for (**a**) A + S; (**b**) A + C; (**c**) G + S and (**d**) G + C using dOA block copolymers under the core contrast system. The symbols are the SANS data, and the solid lines are the model fits.

For more detailed structural information on the C3Ms, a contrast matching technique, i.e., small-angle neutron scattering (SANS), was performed using block copolyelectrolytes with deuterated PEO block (i.e., dOA). Figure 5 displays the SANS data for 0.5 wt % C3Ms of A + S, A + C, G + S, and G + C under the core contrast system. Since SLD_solvent_ is matched to SLD_corona_ of dPEO, the core characteristics are focused such as the aggregation number (*N*_agg_), the core radius (*R*_core_), and the solvent fraction in the core (*f*_sol,core_). SANS data are reproduced by the detailed model (see Appendix A), and representative fitting parameters are listed in Table 2.

Figure 5a–d are representative profiles of the sphere form factor with a smeared, but distinct first minimum. Based on the characteristic equation for the minima in the sphere form factor, sin(*qR*_core_) − *qR*_core_ cos(*qR*_core_) = 0, the core radius can be estimated as *R*_core_ = 8.1, 7.0, 8.9, and 8.3 nm for C3Ms of A + S, A + C, G + S, and G + C, respectively. These estimated *R*_core_ agrees well with the fitting results with the detailed model. In addition, it is striking that simply mixing two oppositely-charged block copolyelectrolytes in an aqueous solution produces relatively monodispersed micelles without the help of organic solvent, based on the value of *σ*_core_/*R*_core_.

Despite the identical degree of polymerization, *R*_core_ and *N*_agg_ are slightly different for the four pairs of ionic groups. C3Ms formed by weaker ionic groups such as A + C give the smallest *R*_core_ and *N*_agg_, but stronger ionic groups such as G + S yield the largest *R*_core_ and *N*_agg_. Intermediate combinations of polyelectrolytes such as A + S or G + C produce C3Ms with intermediate *R*_core_ and *N*_agg_.

In terms of the micelle structure, the unfavorable interaction between the core domain and solvent matrix tends to minimize an interfacial area per chain on the core surface, and to stretch the core block, which results in larger *R*_core_ and *N*_agg_ [59]. However, balanced *R*_core_ and *N*_agg_ for a given block copolymer exist because the stretched block in the core are entropically unfavorable. Based on the theoretical description, it is reasonable that the complex coacervates of strong ionic groups such as G + S shows higher interfacial tension against water than that of weak ionic groups. This also implies that the thermodynamics underlying the formation of complex coacervates are significantly sensitive to the pairs of ionic groups rather than simple charge pairing [11,40,60].

Based on the fitted values, *f*_sol,core_ can be estimated as (1 − *f*_sol,core_)(4/3)π*R*_core_^3^ = *N*_agg_*ν*_core_, where *ν*_core_ is the PAGE block volume (Note that the volume of PAGE block is used to estimate the polymer volume in the cores because the volume of charged block in an aqueous solution is difficult to measure [41,47]). As expected, a large amount of water (*f*_sol,core_ ~ 0.8) is maintained inside the coacervate cores for the four C3Ms, which shows good agreement with previous results [61,62,63,64]. This reflects the fact that the cores of C3Ms are relatively hydrophilic compared to traditional hydrophobic-driven micelles.

**Figure 6:**
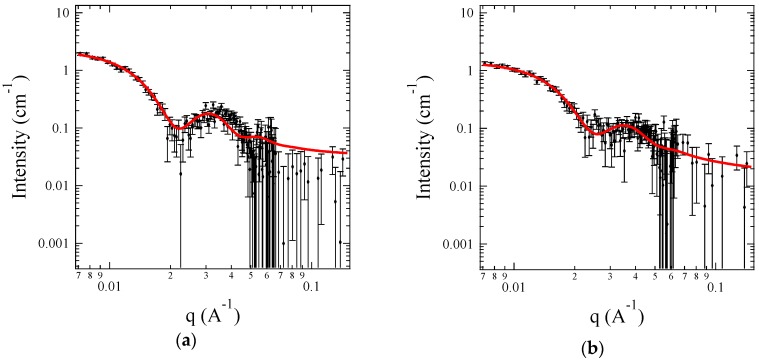

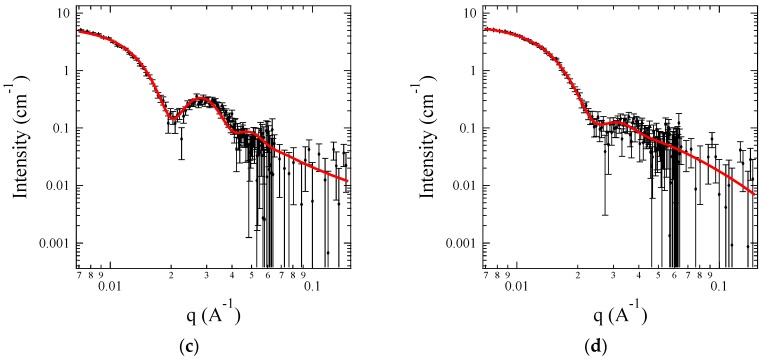
SANS profiles 0.5 wt % C3Ms for (**a**) A + S; (**b**) A + C; (**c**) G + S and (**d**) G + C using dOA block copolymers under the corona contrast system. The symbols are the SANS data, and the solid lines are the model equation fits.

Figure 6 shows the SANS profiles for 0.5 wt % C3Ms of A + S, A + C, G + S, and G + C under the corona contrast system, where SLD_solvent_ is exactly matched to SLD_core_ by mixing H_2_O and D_2_O. Since the core domains are invisible, chains in the corona are focused, such as monomer distribution in the corona, and the radius of gyration of the corona block (*R*_g_). Representative fitting parameters are listed in Table 3. Both *R*_core_ and *N*_agg_ show good agreement with the core contrast system. It is also observed that the corona thickness, roughly estimated by 2 × *R*_g_, is comparable to *R*_core_, even though the core block length is far smaller than the corona block length. This can be understood by the swollen core with a significant amount of water.

Here, *R*_g_ of the corona chain can be compared to the unperturbed <*R*_g_>_0_ of PEO to provide a dimensionless parameter *s*_corona_ (≡*R*_g_/<*R*_g_>_0_), and measure of chain stretching. Since <*R*_g_>_0_ of PEO (*M*_w_ = 10,000 g/mol) in water is calculated as 3.16 nm, *s*_corona_ is 1.2, 1.2, 1.3 and 1.1 for A + S, A + C, G + S and G + C C3Ms, respectively [65,66]. This indicates that PEO chains attached on the core surface are not considerably stretched out. In addition, the corona chain can be characterized by the dimensionless brush density, defined as the ratio of the projected surface area per chain (=π<*R*_g_>_0_^2^) to the available surface area per chain (=4π*R*_core_^2^/*N*_agg_). This is a two-dimensional analog of the reduced concentration *c*/c*, where c* is the overlap concentration in a solution. The dimensionless brush density is calculated as 1.7, 1.5, 2.0, and 2.2 for A + S, A + C, G + S, and G + C C3Ms, respectively, which also indicates that PEO chains are slightly overlapped, and thus not highly stretched. Compared to the traditional block copolymer micelles, the corona chains in C3Ms are relatively relaxed because the cores are highly swollen by water, and thus roomy surface area is available per chain.

The density profile of the corona chains, *ρ*_corona_(*r*), can be estimated from the fitting parameters including *R*_core_, and cubic b spline parameters, *s* and *a*_1_, and rescaled as follows:

(1) $$\int 4\pi \rho_{corona}\left(r\right)r^{2}dr=N_{agg}v_{corona\;block}$$

**Figure 7:**
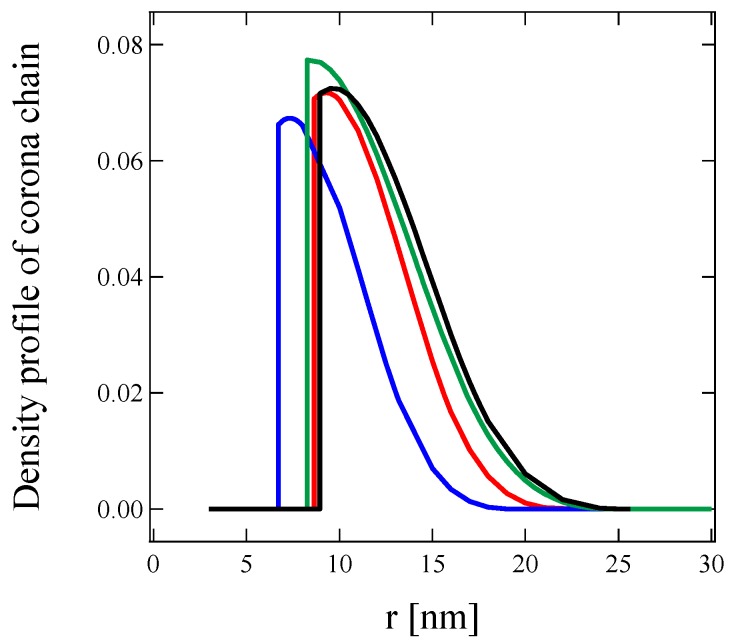
Radial density profile of the corona chains for 0.5 wt % C3Ms of A + S (**red**), A + C (**blue**), G + S (**black**), and G + C (**green**) (color online).

The rescaled *ρ*_corona_(*r*) represents the volume fraction of the corona chains attached to the core surface, and is displayed in Figure 7 for 0.5 wt % C3Ms of A + S, A + C, G + S, and G + C in an aqueous solution. For all C3Ms, the maximum volume fraction of the corona chain is below 0.08, which is significantly lower than traditional block copolymer micelles [58,67,68,69]. The decrease in the maximum of the volume fraction also reflects the dilution of the corona because of the core swelling.

Based on the SANS measurement, the overall micelle dimension can be estimated as *R*_core_ + 2 × *R*_g_ with the assumption of nonpenetration of the PEO chains into the core. Based on Table 3, the overall dimension is 16.26 nm, 14.23 nm, 16.94 nm, and 15.33 nm with narrow distribution for C3Ms of A + S, A + C, G + S, and G + C, respectively. Since the overall dimension obtained from A + S and A + C shows good agreement with *R*_h_ measured by DLS, the C3Ms of A + S and A + C are well-dispersed spherical micelles. However, *R*_h_ is larger than the overall micelle dimension for G + S, and the distribution of *R*_h_ is considerably large for G + S and G + C, which is not consistent with SANS results. Since the combination of TEM images and SANS measurement confirms that individual C3Ms of G + S and G + C are monodispersed spheres, kinetically-trapped structure is suspected where a small fraction of spherical G + S and G + C C3Ms behaves as aggregates to translate cooperatively in the solution. We suspect that both the lower coverage of the PEO shell and the strong electrostatic interaction leads to the kinetically-trapped structure. This implies that the structure and dynamics of C3Ms are considerably sensitive to the pairs of ionic groups, which propose a promising future avenue for experiments.

## 4. Conclusions

In this study, we investigated the structure of complex coacervate core micelles (C3Ms) as a function of the pair of ionic groups including ammonium (A), guanidinium (G), carboxylate (C), and sulfonate (S) using DLS, cryo-TEM, and SANS with a contrast matching technique and a detailed model analysis. C3Ms are formed by the simple mixing of two oppositely-charged block copolyelectrolytes in an aqueous solution. The block copolyelectrolytes were prepared by post-modification of well-defined poly(ethylene oxide-*b*-allyl glycidyl ether) (OA) to keep the degree of polymerization of charged group exactly the same. All C3Ms form spherical micelles with a narrow distribution in the core dimension, and contain large amount of water in the core area, which leads to a loose and relatively relaxed corona layer in the C3Ms. The core radius (*R*_core_) and aggregation number (*N*_agg_) become larger as the interaction between the pair of polyelectrolytes becomes strong (i.e., difference in pKa between the pair), which reflects that the nature of the ion pair plays a key role in the formation of complex coacervation, and thus, C3M. In addition, it is speculated that C3Ms of the pair of strong electrolytes (i.e., G + S) can behave as kinetically-trapped micellar aggregates due to lower coverage of PEO shell and strong electrostatic interaction. Overall, these finding will aid in designing complex fluids based on dispersions of C3Ms in aqueous solutions.

## Figures and Tables

**Table 1:** Polymer Characteristics.

Polymer	*M*_n,PEO_ (kg/mol)	*N*_PEO_*^a^*	*M*_n,PAGE_ (kg/mol)	*N*_PAGE_*^a^*	*Ð*
PEO	10	227	–	–	1.04
dPEO	10	227	–	–	1.07
OA	10	227	5.9	52	1.05
dOA	10	227	5.9	52	1.06

^a^ degree of polymerization of PEO and PAGE block.

**Table 2:** SANS fitting result of core contrast system.

	*R*_core_ (nm)	*σ*_core_ (nm)	*N*_agg_	*f*_sol,core_
A + S	8.56	1.31	50	0.83
A + C	7.58	1.44	35	0.83
G + S	9.50	1.60	72	0.82
G + C	8.86	1.40	68	0.79

**Table 3:** SANS fitting result of corona contrast system.

	*R*_core_ [nm]	*σ*_core_ (nm)	*N*_agg_	*R*_g_ (nm)	*a*_1_	*s* (nm)
A + S	8.56	1.18	50	3.85	–0.41	6.98
A + C	6.71	1.12	30	3.76	–0.43	6.50
G + S	8.94	1.17	71	4.00	–0.40	8.34
G + C	8.27	0.91	68	3.53	–0.08	8.57

## Supplementary Materials

The following are available online at https://www.mdpi.com/2073-4360/11/3/455/s1, Figure S1: GPC traces of (a) PEO and dPEO homopolymer and (b) OA and dOA block copolymer in chloroform., Figure S2: ^1^H-NMR spectra of OA block copolymer, Figure S3: ^1^H-NMR spectra of functionalized OA with Ammonium (A, –NH_3_^+^), Guanidinium (G, –NHCNHNH_3_^+^), Carboxylate (C, –COO^−^), and Sulfonate (S, –SO_3_^−^), Figure S4: SANS profiles of 0.5 wt % A + C C3Ms in an isotopic mixture of H_2_O and D_2_O with various fraction of D_2_O, Figure S5: SANS intensity of C3Ms as a function of D_2_O fraction for (a) A + S, (b) A + C, (c) G + S, and (d) G + C, Figure S6: SAXS profiles of 0.5 wt % C3Ms for (a) A + S, (b) A + C, (c) G + S and (d) G + C using dOA block copolymers in H_2_O. The symbols are the SAXS data and the solid lines are the model fits. Here, a core-shell sphere with polydisperse core model is employed, Table S1: Estimated SLD_core_ for the C3Ms, Table S2: Fitting results of C3Ms.

## Appendix A

### Fitting Model

To fit the SANS data for C3M, the form factor for block copolymer micelles developed by Pedersen et al. was used [70,71]. The fitting model assumes (1) spherical cores with a gaussian distribution in core radius, (2) corona PEO chains attached to the core surface, and (3) a liquid-like interaction between hard spheres.

The form factor involves four terms: self-correlation of core or corona, cross term between core and corona, and cross term between different corona chains.

(A1) $$\begin{array}{ll} F\left(q\right)= & N_{agg}^{2}\beta_{core}^{2}A_{core}^{2}\left(q\right)+N_{agg}\beta_{corona}^{2}P_{chain}\left(q\right)\\ & +2N_{agg}^{2}\beta_{core}\beta_{corona}A_{core}\left(q\right)A_{corona}\left(q\right)\\ & +N_{agg}\left(N_{agg}-1\right)\beta_{corona}^{2}A_{corona}^{2}. \end{array}$$

where *N*_agg_ is an aggregation number of the micelle, and *β*_core_ and *β*_corona_ is total excess scattering lengths of the core and corona. They are defined as *β*_core_ = *v*_core_(SLD_core_ − SLD_solvent_) and *β*_corona_ = *v*_corona_ (SLD_corona_ − SLD_solvent_), where *v*_core_ and *v*_corona_ are the volumes of core and corona blocks, respectively, and *SLD*_core_, *SLD*_corona_ and *SLD*_solvent_ are the scattering length density of core and corona block and solvent, respectively.

First term indicates the self-correlation of the homogenous spherical cores with smooth decay in scattering length density at the core-corona interface.

(A2) $$A_{core}^{2}\left(q\right)=\Phi^{2}\left(qR_{core}\right)e^{\left(-q^{2}\sigma_{int}{}^{2}\right)}$$

where Φ(*x*) is the hard-sphere form factor, defined as Φ(*x*) = 3[sin*x* − *x*cos*x*]/*x*^3^, *R*_core_ is the homogeneous core radius, and *σ*_int_ is the width of the interface between core and corona.

Second term indicates the self-correlation term of corona chain with radius of gyration, *R*_g_, which can be described as Debye function.

(A3) $$P_{chain}\left(q\right)=\frac{2\left[\exp\left(-q^{2}R_{g}^{2}\right)-1+q^{2}R_{g}^{2}\right]}{{\left(q^{2}R_{g}^{2}\right)}^{2}}$$

The last two terms in eq A1 are the cross term between core and corona and between corona chains. Both terms contain the form factor of the corona chains, *A*_corona_(*q*), that is given as the normalized Fourier transform of the radial density distribution function of the corona chain, *ρ*_corona_(r).

(A4) $$A_{corona}\left(q\right)=\frac{4\pi \int \rho_{corona}\left(r\right)\frac{\sin\left(qr\right)}{qr}r^{2}dr}{4\pi \int \rho_{corona}\left(r\right)r^{2}dr}\exp\left(\frac{-q^{2}\sigma_{int}^{2}}{2}\right)$$

where *r* is the distance from the core-corona interface. In this study a linear combination of two cubic b spline functions is selected as *ρ*_corona_(r), which has been successfully applied to describe the micelle structure [41,72].

Based on the form factor, the total coherent scattering intensity is given as

(A5) $$I\left(q\right)=F\left(q\right)+A_{mic}{\left(q\right)}^{2}\left[S\left(q\right)-1\right]$$

where *A*_mic_ is the amplitude of form factor of the radial scattering length distribution of the micelles, which is defined as *A*_mic_(*q*) = *N*_agg_(*β*_core_ *A*_core_ (*q*) + *β*_corona_ *A*_corona_(*q*)), and *S*(*q*) is the structure factor for randomly dispersed hard spheres [41,72].

In addition, distribution in core size is considered by Gaussian distribution function of *D*(*R*_core_).

(A6) $$D\left(R_{\mathrm{core}}\right)=\frac{1}{\sqrt{2\pi }\sigma_{core}}\exp\left(\frac{-{\left(R_{\mathrm{core}}-R_{\mathrm{core}}\right)}^{2}}{2\sigma_{core}{}^{2}}\right)$$

where *σ**_core_* is the width of distribution of the core size and <*R*_c__ore_> is average core size. Consequently, the fitting model with polydisperse hard sphere is expressed as shown below.

(A7) $$I\left(q\right)=\int D\left(R_{core}\right)\left(F\left(q\right)+A_{mic}{\left(q\right)}^{2}\left[S\left(q\right)-1\right]\right)dR_{core}$$
