# Supplementary material for: Effect of Ionic Group on the Complex Coacervate Core Micelle Structure
Source: Polymers (Basel). 2019 Mar 10;11(3):455. doi: 10.3390/polym11030455 (PMC6473896; doi:10.3390/polym11030455)
Supplement: Supplementary file 1 [file polymers-11-00455-s001.pdf]

# Supporting Information

## Effect of Ionic Group on the Complex Coacervate Core Micelle Structure

Tae-Young Heo[1], Inhye Kim[2], Liwen Chen[3], Eunji Lee[4], Sangwoo Lee[3], Soo-Hyung Choi*,[1]

[1] Department of Chemical Engineering, Hongik University, Seoul, 04066, Rep. of Korea

[2] Graduate School of Analytical Science and Technology, Chungnam National University, Daejeon, 34134, Rep. of Korea

[3] Department of Chemical & Biological Engineering, Rensselaer Polytechnic Institute, Troy, NY, 12180, USA

[4] School of Materials Science and Engineering, Gwangju Institute of Science and Technology, Gwangju, 61005, Rep. of Korea

*Author for correspondence: shchoi@hongik.ac.kr

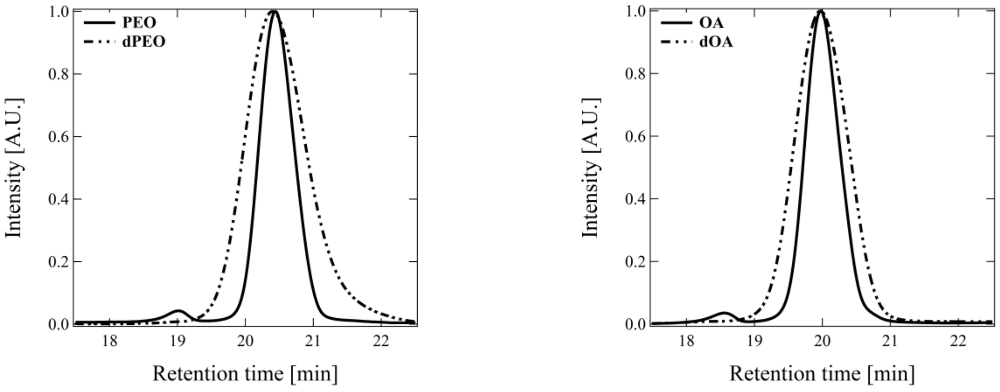


(a) (b)

**Figure S1.** GPC traces of (a) PEO and dPEO homopolymer and (b) OA and dOA block copolymer in chloroform.

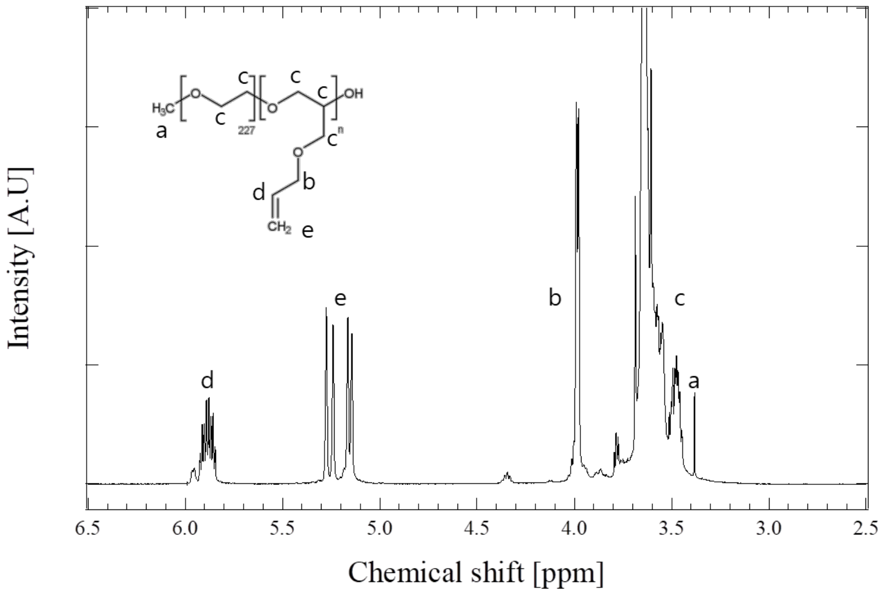


**Figure S2.** $^{1}$H-NMR spectra of OA block copolymer:
$^{1}$H-NMR (400MHz, $CDCl_3$, δ ): 3.38 (s, -OC$H_3$), 3.40-3.80 (-OC$H_2$C$H_2$O-, -OC$H_2$C$H$C$H_2$-), 4.00 (d, -OC$H_2$CH=$CH_2$), 5.15 (d, -O$CH_2$CH=C$H_2$), 5.25 (d, -O$CH_2$CH=C$H_2$), 5.90 (m, -O$CH_2$C$H$=$CH_2$).

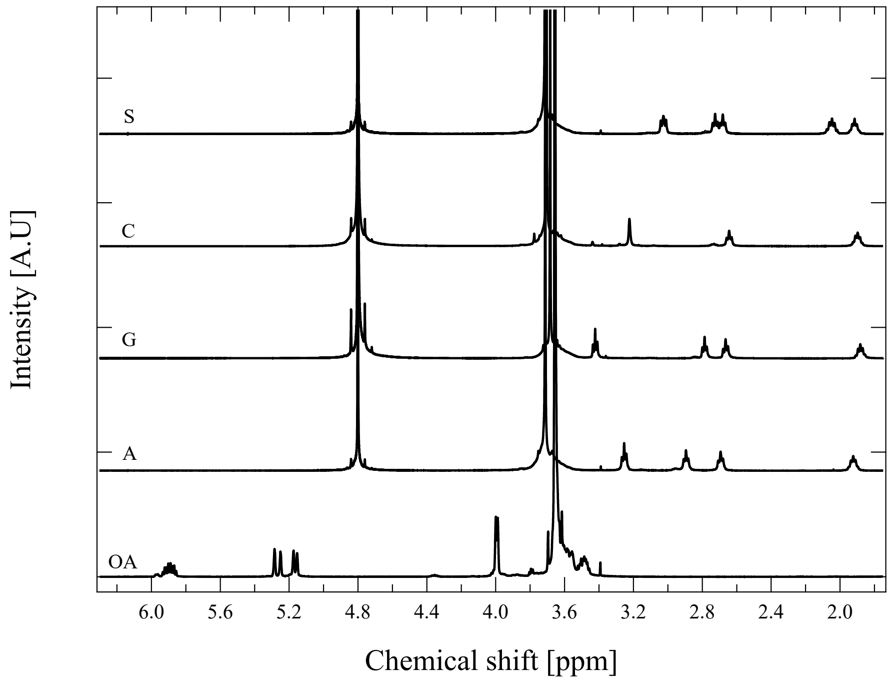


**Figure S3.** $^1$H-NMR spectra of functionalized OA with Ammonium (A, -$NH_3^+$), Guanidinium (G,-$NHCNHNH_3^+$), Carboxylate (C,-$COO_3^-$), and Sulfonate (S, -$SO_3^-$):

**A**: $^1$H-NMR (400MHz, $D_2O$, δ) : 1.92 (-$CH_2C\textit{H}_2CH_2$-), 2.69 (-$CH_2CH_2C\textit{H}_2S$), 2.89 (-$SC\textit{H}_2CH_2NH_3^+Cl^-$), 3.25 (-$SCH_2C\textit{H}_2NH_3^+Cl^-$), 3.5-4.0 (-$OC\textit{H}_2C\textit{H}_2O$-, -$OC\textit{H}_2C\textit{H}$-, -$OC\textit{H}_2CH_2$-).

**G**: $^1$H-NMR (400MHz, $D_2O$, δ) : 1.92 (-$CH_2C\textit{H}_2CH_2$-), 2.69 (-$CH_2CH_2C\textit{H}_2S$), 2.79 (-$SC\textit{H}_2CH_2NH$), 3.42 (-$SCH_2C\textit{H}_2NH$), 3.5-4.0 (-$OC\textit{H}_2C\textit{H}_2O$-, -$OC\textit{H}_2C\textit{H}$-, -$OC\textit{H}_2CH_2$-).

**C**: $^1$H-NMR (400MHz, $D_2O$, δ) : 1.91 (-$CH_2C\textit{H}_2CH_2$-), 2.64 (-$CH_2CH_2C\textit{H}_2S$ ), 3.22 (-$SC\textit{H}_2COO^-Na^+$), 3.5-4.0 (-$OC\textit{H}_2C\textit{H}_2O$-, -$OC\textit{H}_2C\textit{H}$-, -$OC\textit{H}_2CH_2$-).

**S**: $^1$H-NMR (400MHz, $D_2O$, δ) : 1.91 (-$CH_2C\textit{H}_2CH_2$-), 2.04 (-$C\textit{H}_2SO_3^-Na^+$), 2.68 (-$CH_2CH_2C\textit{H}_2S$), 2.73 (-$SC\textit{H}_2CH_2CH_2SO_3^-Na^+$), 3.03 (-$SCH_2C\textit{H}_2CH_2SO_3^-Na^+$), 3.5-4.0 (-$OC\textit{H}_2C\textit{H}_2O$-, -$OC\textit{H}_2C\textit{H}$-, -$OC\textit{H}_2CH_2$-).

**Scattering length density (SLD) of C3M cores**

In order to investigate more detailed structure of the C3M using small angle neutron scattering (SANS), the scattering length density of the core ($SLD_{core}$) is required. Since it is assumed that the core domains are formed by two oppositely charged blocks, the $SLD_{core}$ for the denser core domain was experimentally measured.

Figure S4 displays SANS patterns for 0.5 wt% A+C C3Ms in an isotopic mixture of $H_2O$ and $D_2O$ with various fraction of $D_2O$. Since the SANS intensity is related as $I \sim (SLD_{core} - SLD_{solvent})^2$, the intensity is reduced as $SLD_{core}$ approaches to $SLD_{solvent}$. Interpolation of the SANS intensity between 0.0066 $Å^{-1} < q <$ 0.011 $Å^{-1}$ provides that $SLD_{core}$ is matched to $SLD_{solvent}$ when the fraction of $D_2O$ is 0.24 as shown in Figure S5(a). Therefore, $SLD_{core}$ for A+C C3M is determined as $1.34 \times 10^{-6}$ $Å^{-2}$. With the same process as shown in Figure S5, $SLD_{core}$ for A+S, G+S, and G+C were determined, and listed in Table S1.

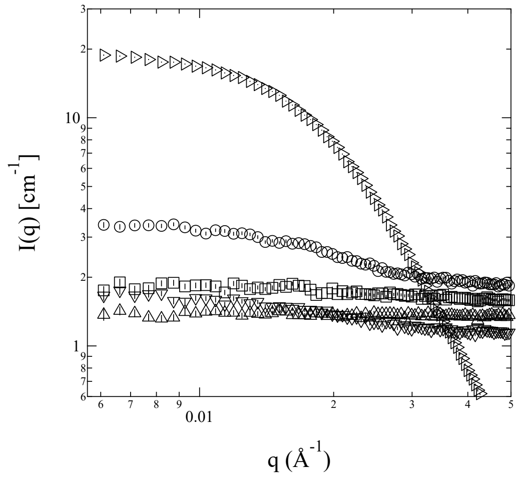


**Figure S4.** SANS profiles of 0.5 wt% A+C C3Ms in an isotopic mixture of $H_2O$ and $D_2O$ with various fraction of $D_2O$: 1 (▷), 0.36 (▽), 0.24 (△), 0.12 (□), and 0 (○).

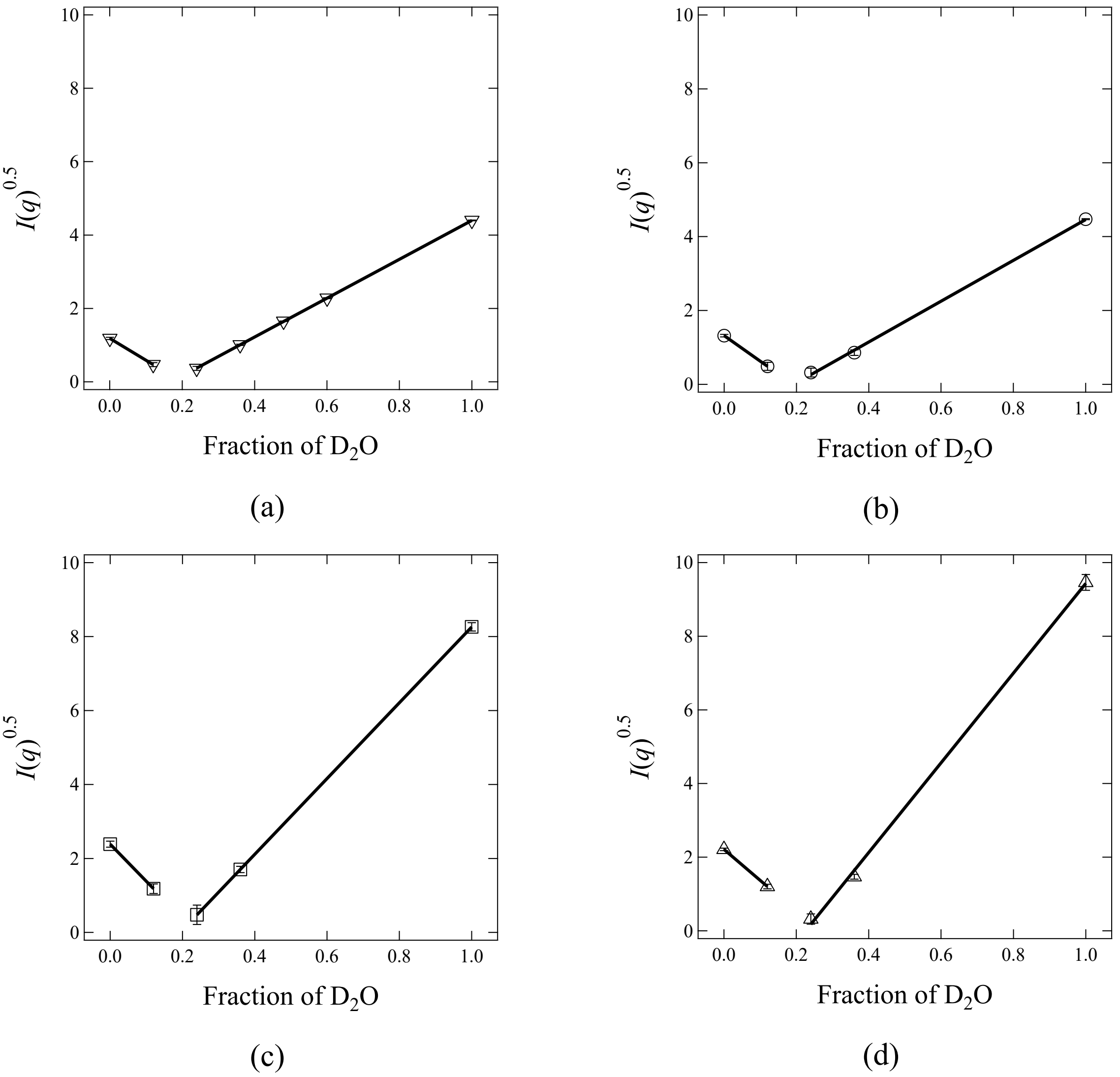


**Figure S5.** SANS intensity of C3Ms as a function of $D_2O$ fraction for (a) A+S, (b) A+C, (c) G+S, and (d) G+C.

**Table S1.** Estimated $SLD_{core}$ for the C3Ms

| | A+S | A+C | G+S | G+C |
|---|---|---|---|---|
| $SLD_{core}$ [$\times 10^{-6}$ Å$^{-2}$] | 1.31 | 1.34 | 1.55 | 1.75 |

## SAXS analysis of C3Ms

SAXS measurement was performed on beamline 4C SAXS at Pohang Acceleration Laboratory (PAL) using 16.9 keV radiation corresponding to a wavelength, $\lambda$, of 0.734 Å. The sample-to-detector distance (SDD) was 4.3 m to cover $q$ range of 0.007 Å$^{-1}$ < $q$ < 0.12 Å$^{-1}$. The C3M solutions were loaded and sealed into boron-rich capillary tubes, and followed by exposure to X-ray for c.a. 1 min. Two-dimensional images were azimuthally averaged to provide one-dimensional plot of intensity $I(q)$ versus $q$. Solvent background was subtracted from the solution scattering.

Since TEM images show that C3Ms in this study are sphere, the SAXS profile was reproduced by the core-shell sphere model with polydispersed core radius as shown below.

$$I(q) = \left[\frac{3 \times V_{core}(\rho_{core} - \rho_{shell})\,\Phi(qR_{core})}{qR_{core}} + \frac{3 \times V_{shell}(\rho_{shell} - \rho_{solvent})\,\Phi(qR_{shell})}{qR_{shell}}\right]^2$$

,where

$$\Phi(x) = \frac{sinx - xcosx}{x^2}$$

where $V_{core}$, $V_{shell}$, $R_{core}$ and $R_{shell}$ are the volume and radius of core and shell, respectively, and $\rho_{core}$, $\rho_{shell}$ and $\rho_{solvent}$ are the electron density of the core, shell and solvent, repectively. In addition, Schulz distribution of the core radius was employed to the model, which accounts for the polydisperse core radius. The model fit shows good agreement with the experimental data as shown in Figure S6, and the adjusted values are shown in Table S2. Both $R_{\mathrm{core}}$ and $\sigma_{\mathrm{core}}$ obtained from SAXS analysis are consistent with the SANS results.

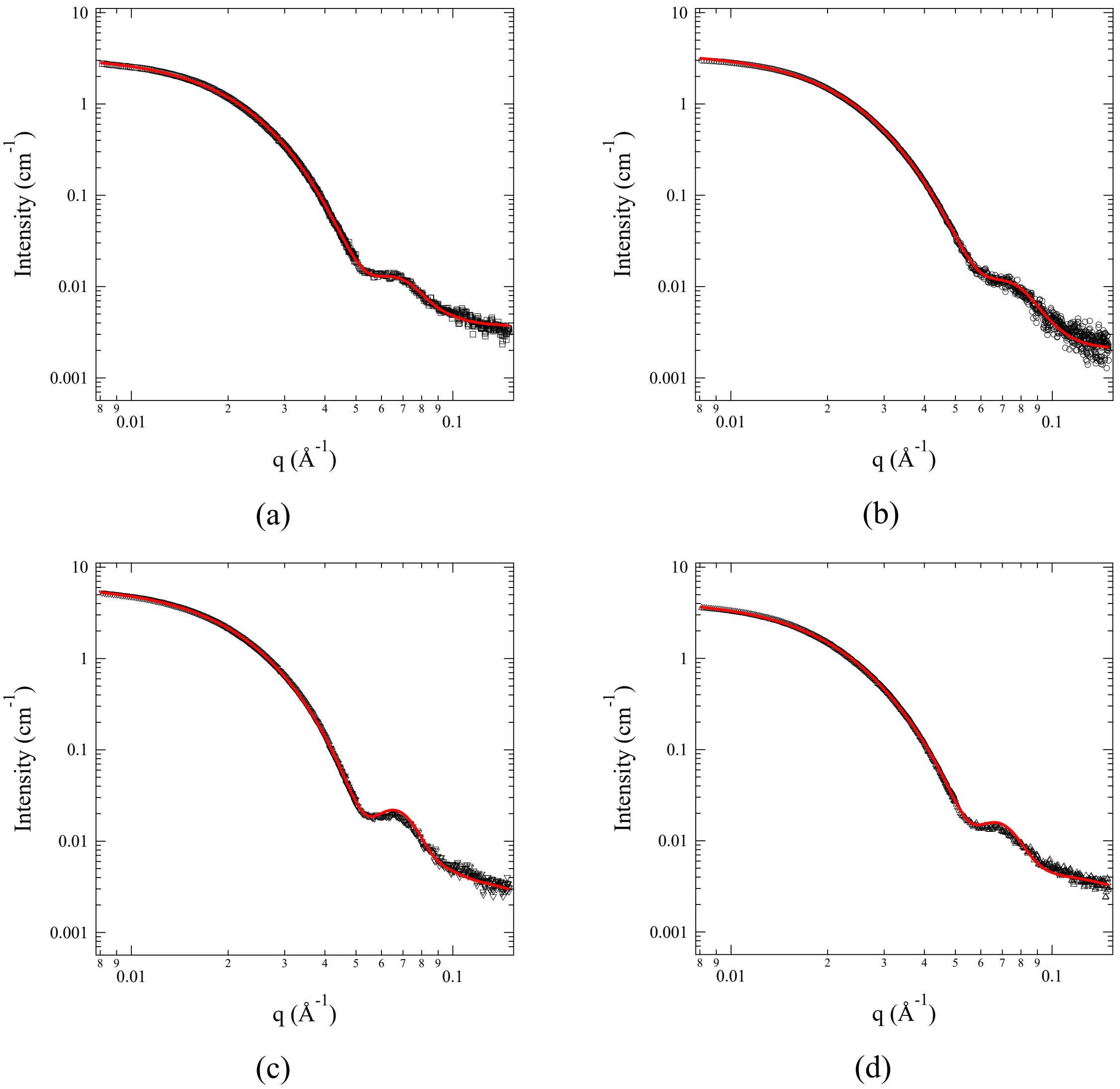


**Figure S6.** SAXS profiles of 0.5wt% C3Ms for (a) A+S, (b) A+C, (c) G+S and (d) G+C using dOA block copolymers in $H_2O$. The symbols are the SAXS data and the solid lines are the model fits. Here, a core-shell sphere with polydisperse core model is employed.

**Table S2.** Fitting results of C3Ms

| | $R_{core}$ [nm] | $\sigma_{core}$ / $R_{core}$ | $R_{shell}$ |
|---|---|---|---|
| A+S | 7.97 | 0.16 | 8.26 |
| A+C | 7.14 | 0.18 | 7.75 |
| G+S | 8.22 | 0.13 | 8.85 |
| G+C | 7.85 | 0.14 | 9.18 |
